# Agrin-Mediated Cardiac Regeneration: Some Open Questions

**DOI:** 10.3389/fbioe.2020.00594

**Published:** 2020-06-16

**Authors:** Maria Giulia Bigotti, Katie L. Skeffington, Ffion P. Jones, Massimo Caputo, Andrea Brancaccio

**Affiliations:** ^1^Bristol Heart Institute, Research Floor Level 7, Bristol Royal Infirmary, Bristol, United Kingdom; ^2^School of Biochemistry, University of Bristol, Bristol, United Kingdom; ^3^Institute of Chemical Sciences and Technologies “Giulio Natta” (SCITEC)—CNR, Rome, Italy

**Keywords:** heart regeneration, agrin, dystrophin-glycoprotein complex, dystroglycan, laminin, YAP, Hippo pathway, cardiomyocyte proliferation

## Abstract

After cardiac injury, the mammalian adult heart has a very limited capacity to regenerate, due to the inability of fully differentiated cardiomyocytes (CMs) to efficiently proliferate. This has been directly linked to the extracellular matrix (ECM) surrounding and connecting cardiomyocytes, as its increasing rigidity during heart maturation has a crucial impact over the proliferative capacity of CMs. Very recent studies using mouse models have demonstrated how the ECM protein agrin might promote heart regeneration through CMs de-differentiation and proliferation. In maturing CMs, this proteoglycan would act as an inducer of a specific molecular pathway involving ECM receptor(s) within the transmembrane dystrophin-glycoprotein complex (DGC) as well as intracellular Yap, an effector of the Hippo pathway involved in the replication/regeneration program of CMs. According to the mechanism proposed, during mice heart development agrin gets progressively downregulated and ultimately replaced by other ECM proteins eventually leading to loss of proliferation/ regenerative capacity in mature CMs. Although the role played by the agrin-DGC-YAP axis during human heart development remains still largely to be defined, this scenario opens up fascinating and promising therapeutic avenues. Herein, we discuss the currently available relevant information on this system, with a view to explore how the fundamental understanding of the regenerative potential of this cellular program can be translated into therapeutic treatment of injured human hearts.

## Introduction

The repair of damaged hearts represents one of the most critical challenges in cardiovascular medicine, since mammalian cardiomyocytes (CMs) lose their proliferative potential very early in life, after which point hypertrophic processes become responsible for most of the remaining heart growth. Therefore, upon injury, CMs are replaced by fibrotic tissue, often with deleterious consequences. Recent advances in understanding the endogenous program(s) regulating the proliferation of cardiomyocytes at the molecular level [reviewed in Deshmukh et al. (2019), Heallen et al. (2019), Ali et al. (2020)] hold great promise for the development of new approaches to heart regeneration based on cell cycle re-activation. Unfortunately, a full characterization of the developmental pathway(s) of the mammalian heart and specifically of CMs, is hindered by an intrinsic difficulty in understanding the exact timing and interchange of proliferation and differentiation events (Velayutham et al., 2019). Unlike certain fish and amphibian species (Zhang et al., 2013), mammalian CMs can follow a regenerative program only during a limited postnatal period. Due to important differences in the developmental milestones of specific mammalian species, the exact time frame of fetal/postnatal proliferative heart growth has however only been determined in few model systems. The best characterized of these is the mouse model, in which full proliferation of CMs lasts for only the first few days postnatally, possibly until postnatal day 2 (P2), with the regenerative program already shut down at P7, when CMs have stopped proliferating and switched to a hypertrophic program (Porrello et al., 2011; Notari et al., 2018). Some recent crucial findings in mice challenge in part the idea that this differentiation program cannot be somehow modulated, and proliferation re-activated (Bassat et al., 2017; Morikawa et al., 2017). Following previous work proving how the rigidity of the extracellular matrix (ECM) strongly influences heart development (Yahalom-Ronen et al., 2015), and specifically CMs maturation, Bassat et al. used a series of elegant experiments to identify the proteoglycan agrin as an ECM component that promotes cardiomyocyte proliferation. Interestingly, they found that this protein is involved in neonatal heart repair and is only enriched in the matrix in the very first postnatal days, while downregulated by the P7 stage. Crucially, administration of recombinant agrin to juvenile and adult infarct mouse models was sufficient to boost cardiac regeneration through reactivation of the CMs proliferative program.

**Figure 1:**
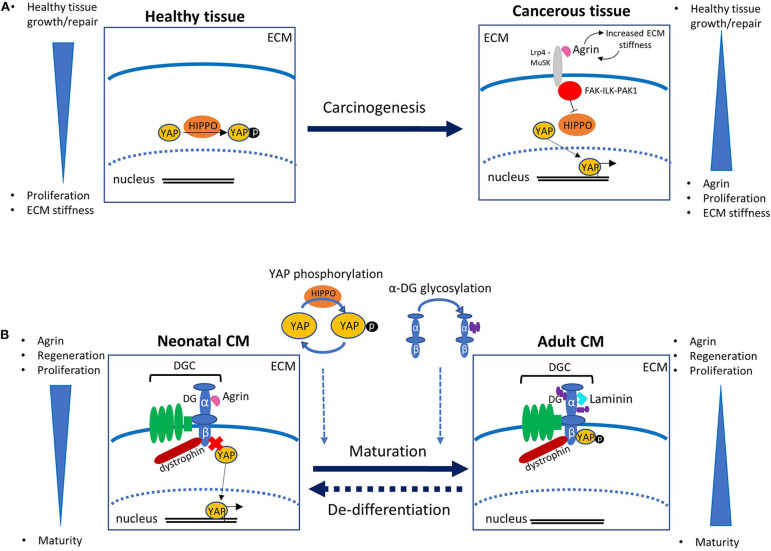
**(A)** Role of agrin and YAP in carcinogenesis. In healthy tissue, the Hippo signaling pathway regulates transcription via phosphorylation of YAP, which results in its confinement out of the cell nucleus. In cancerous tissue an increase in ECM stiffness is associated with increased levels of extracellular agrin. Agrin binds to the receptor Lrp4, which activates MuSK, eventually promoting a signaling cascade involving the FAK-ILK-PAK1 axis. This results in the inhibition of the Hippo pathway that in turn leads to an increase in unphosphorylated YAP. The activity of YAP in the nucleus promotes not only cell-cycle and division but also a further increase in ECM stiffness, possibly following enhanced agrin expression, in a positive feedback mechanism that leads to uncontrolled proliferation. **(B)** Schematic of the role of agrin in mouse cardiomyocyte (CM) proliferation as proposed in Bassat et al. (2017). In neonates, binding of agrin to the α-subunit of dystroglycan (DG) interferes with its correct assembly within the dystrophin-glycoprotein complex (DGC), and ultimately prevents the β-subunit from binding the transcription factor YAP, whose activity in the nucleus promotes CM proliferation. With maturation, replacement of agrin with a different protein (probably laminin) of the extracellular matrix (ECM) allows for a stable assembly of the DGC, so that β-DG can bind and sequester phosphorylated YAP out of the nucleus, thus eventually hindering CM proliferation. The timing and mechanism of YAP phosphorylation, as well as of α-DG glycosylation, have to be determined yet, and might have a role in the molecular pathway. The dashed arrow between the two panels indicates the potential re-activation of CM proliferation induced by controlled delivery of exogenous agrin to injured cardiac tissue.

Existing studies investigating the role of agrin in other biological organs and systems are invaluable for beginning to piece together the mechanisms of action of agrin in the heart. In recent years, agrin has been revealed to play an important role in cancer development. An increase in agrin has been demonstrated in cancerous cells, and studies using experimental models have shown that addition of agrin promotes cancer whilst agrin depletion slows tumor growth (Chakraborty et al., 2015, 2017; Lv et al., 2017; Rivera et al., 2018). It is now known that agrin forms a mechanotransductive link between the ECM and the transcriptional co-activators YAP (Yes associated protein) and TAZ (transcriptional activator with PDZ binding motif) [reviewed in Chakraborty and Hong (2018), Xiong and Mei (2017)]. YAP and TAZ activate the transcription of many pro-proliferative and anti-apoptopic genes and are themselves controlled by many upstream factors, including the Hippo signaling pathway which regulates growth via YAP phosphorylation, thus sequestering it in the cytosol and preventing its transcriptional activity (Figure 1A). Experiments using liver tumor cells suggest that the increase in ECM stiffness which is observed in at least some cancerous tissues results in increased levels of extracellular agrin (Chakraborty et al., 2017). Agrin binds to the cell via Lrp4 (low density lipoprotein receptor related protein 4), which in turn activates MuSK (muscle specific kinase 4). This results in activation of the FAK-ILK-PAK1 (Focal Adhesion Kinase—Integrin Linked Kinase—p21 Activated Kinase) signaling axis, resulting in inhibition of Hippo signaling and consequent nuclear localization of YAP/TAZ. YAP transcriptional activity itself is known to increase the ECM stiffness possibly due to enhanced agrin expression, and it has been suggested that a positive feedback loop thus exists between agrin levels (through ECM stiffness increase) and YAP transcriptional activity, in a functional mechanotransduction network that promotes uncontrolled proliferation (Calvo et al., 2013; Chakraborty and Hong, 2018) (Figure 1A). Similar signaling pathways exists in neuromuscular junctions, where agrin is responsible for recruiting acetylcholine receptors. Agrin secreted from nerve terminals activates YAP via Lrp4/MuSK signaling; this results in the aggregation of acetylcholine receptors on the postsynaptic muscle cells (Zhao et al., 2017; Chakraborty and Hong, 2018). Agrin has also been demonstrated to interact with dystroglycan (DG) at the neuromuscular junction; DG is a pivotal member of the large transmembrane complex connecting the ECM with the cytoskeleton [namely the dystrophin-glycoprotein complex (DGC)], which is necessary for stabilization of acetylcholine receptor clusters (Ervasti and Campbell, 1993; Jacobson et al., 2001). In line with these precedents, a role for agrin in similar pathways have been demonstrated to exist in the heart. Bassat et al. determined that agrin promotes heart regeneration by interacting with the DGC on the CMs cell surface, thus preventing its assembly into the stable adult form. Specifically, the interaction of agrin with the extracellular α-DG on CMs would influence the binding properties of the transmembrane/cytoplasmic β-subunit of DG, favoring its binding to dystrophin over that to YAP. Similarly to what described for cancer growth, YAP is known to be involved in heart growth through CM proliferation which, unlike in other tissues, is inhibited postnatally due to the activity of upstream kinases of the Hippo pathway (Von Gise et al., 2012; Xin et al., 2013). The view proposed by Bassat et al. was corroborated and complemented by equally important findings from the James Martin's laboratory which showed how, during heart maturation, YAP gets phosphorylated by the Hippo pathway and consequently establishes a stable interaction with the cytodomain of β-DG in the adult tissues (Morikawa et al., 2017). Such interaction would sequester YAP at the cell periphery, thus preventing it from exerting its proliferation-inducing activity in the nucleus. All this evidence has been put together to envisage a possible molecular mechanism linking the axis agrin-DGC-YAP to heart tissue proliferation, in which during development and very early after birth the interaction of agrin with α-DG would interfere with the proper assembly of β-DG into the DGC, ultimately preventing the tethering of YAP. During cardiac maturation, this arrangement would change since on one hand YAP would get actively phosphorylated by the Hippo signaling pathway, on the other agrin would be replaced by a different, yet unidentified, ECM component (see below), thus allowing for proper DGC assembly. These two events would lead to the locking of phospho YAP at the inner side of the cell surface through β-DG binding (Figure 1B). A series of evidences shows how, in this proposed mechanism, laminin represents a strong candidate to take over from agrin in the binding to the DGC axis during cardiac muscle development, at least at the later stages. It was in fact shown that embryonic stem cells carrying mutations in laminin 211, although differentiating normally *in vitro* into contracting myotubes, eventually undergo detachment and death (Kuang et al., 1998). Together with the evidence that mutations or complete lack of laminin 211 are known to cause severe forms of congenital muscular dystrophy (CMD), these results stress the important role of laminin for adult skeletal muscle stabilization (Yurchenco et al., 2018) and cardiac muscle stability (Nguyen et al., 2019). Other laminins too (including laminin 511, 521, and 221) are known to play an important role for the differentiation of stem cells into cardiomyocytes (Wang et al., 2019; Yap et al., 2019; Chanthra et al., 2020). Recent evidence from studies in *Drosophila* suggests the possible involvement of β-DG in the Hippo signaling pathway as a mechano-receptor within muscle cells (Yatsenko et al., 2020), thus it is worth noting that the DG-Hippo pathway axis might have an evolutionarily conserved role in mediating muscle growth and regeneration. Although it is well-known that the composition of cardiac ECM, and thus its mechanosensing properties, play a fundamental role in heart development (Parker and Ingber, 2007; Garoffolo and Pesce, 2019), whether agrin regulates cardiac ECM stiffness, similarly to its role in hepatic cancer cells, is unknown. It will be certainly worth to investigate this point further, in order to understand whether, irrespective of the ECM receptors involved, agrin employs a similar strategy to stimulate proliferation of CMs and cancer cells.

## A New Hope for Cardiac Regeneration in Mammals?

Aside from the fact that several aspects of the proposed mechanism remain unclear (see some of the points below), the fundamental question arising is whether a similar mechanism is employed also by human heart. A pretty tricky question indeed, in the face of the complex and still elusive task of establishing a correlation between the developmental stages of different mammals that has to be added to the intrinsic challenge posed by development studies of the human heart. With a focus on the molecular aspects and on the therapeutic translational potential of agrin in human medicine, we have tried to highlight and discuss some of the open points (summarized in Box 1).

Box 1 **Open questions on the cardiac regenerative role of the agrin-DG-Yap axis.**

**Open questions:**

- How is the balance between agrin and laminin in binding to their receptor α-DG controlled/modulated?
- What is the impact of this agrin-dependent mechanism on CM proliferation during mouse heart growth?
- Does a similar pathway exist in human cardiac development?
- If so:
  1. What is the exact timeframe of the switch in humans?
  2. What is the contribution of the agrin-driven pathway to CM proliferation and to the overall development of human heart?
  3. Can we harness this pathway therapeutically to treat cardiac injury in humans without deleterious effects?

### Role of ECM in Cardiac Regeneration: Agrin vs. Laminin, how Would Molecular Specificity Be Controlled?

The data collected by Bassat et al. (2017) using mouse models led them to propose that the regenerative program of CMs is directly linked to the levels of agrin in the ECM, which decrease over time to a minimum reached by P7 (likely earlier), when CMs have already switched to the hypertrophic program. Following this line of enquiry, the time window was revealed to be even shorter. Recent mouse experiments employing heart apical amputation as a tool to analyze possible regenerative pathways (Notari et al., 2018) established in fact that the timing at which CMs full proliferative program(s) can be triggered is even tighter than the postnatal period P1-P7 previously reported (Porrello et al., 2011, 2013). Indeed, a high degree of regeneration was observed only at P1, with the speed lowering considerably at P2–P3. This would suggest that the window available for fully inducing massive CM proliferation could be as short as 24 h after birth. Based on an overall transcriptomic analysis, the same study proposes that extracellular matrix proteins, rather than intracellularly acting cardiac fibers or transcription factors, are the most likely to influence this process by affecting/regulating the mechanical degree of stiffness. This would point to a predominantly “outside-inside” nature of the overall proliferation program, similarly to what has been shown in cancer cells, although the molecular mechanisms underlying it are yet to be elucidated.

The work of Bassat et al. clearly shows that the levels of agrin in mice are halved at P7, although how the protein changes between P1 and P7 is still unknown. As a matter of fact, the exact timing at which agrin ceases to have a measurable effect on the regeneration program remains to be debated. The only available data on the very first days after birth are those of Notari et al., and show how the expression levels of agrin (and its receptor dystroglycan) do not change between P1 and P2 [see Notari et al. (2018), Figure S5C]. With regard to the role of the agrin axis, there are two possible explanations for the lack of regenerative potential already observed at P2: (i) if it is laminin that replaces agrin during heart maturation, it could become over-expressed by P2 and therefore make a take-over on agrin because they display a similar affinity toward α-DG [see Sciandra et al. (2013)], (ii) post-P2, additional factors could intervene that favor the binding of laminin to α-DG over agrin. Notably, western blots reveal the presence of laminin already at P1 but a slight raise (~1.3 times) of its levels at P2 (Notari et al., 2018), so not only could either hypothesis be correct, but the two mechanisms could coexist.

From the DGC receptor point of view, a specific molecular aspect that plays an important role in the binding to α-DG is the degree of glycosylation of α-DG itself. For example, in some dystroglycanopathies a significant reduction of α-DG glycosylation (hypoglycosylation) leads to a drop in laminin binding affinity, inducing severe muscular dystrophy phenotypes such as Walker-Warburg Syndrome or Muscle-Eye-Brain disease (Falsaperla et al., 2016; Brancaccio, 2019). It is known that cardiac α-DG is slightly less glycosylated than the skeletal muscle one (Brancaccio et al., 1995) and more glycosylated than brain α-DG (McDearmon et al., 2006). However, in chickens the affinity of cardiac α-DG to laminin-111 is still very high (Brancaccio et al., 1995), and agrin has only a slightly reduced affinity for brain DG relative to skeletal muscle, kidney, or lung (Gesemann et al., 1998). Overall, these data suggest that the effect of differential tissue glycosylation of α-DG on its interaction with both laminin and agrin might be small. Nonetheless, considering that the glycosylation shell of brain α-DG is similar to that of early embryonic α-DG (Leschziner et al., 2001), it may well be that agrin and laminin could display some minor although physiologically relevant differences in affinity toward α-DG tissue isoforms based on their differential degree of glycosylation at various stages of development. To the best of our knowledge no data on this are available in the literature, but if that was the case, it would be important to investigate with some direct protein analysis whether and when there is a transition from cardiac embryonic (less-glycosylated) to cardiac adult (more-glycosylated) α-DG in cardiac myocytes and fibers. From a translational point of view, such knowledge could guide the optimization of exogenous recombinant agrin that preferentially interacts with fully glycosylated α-DG over laminin in differentiated CMs, as to increase its regenerative potential upon administration to damaged hearts.

Although unlikely, a further scenario that should be considered is the possible presence of an additional “factor” that would be switched on after P2 to freeze/lock the system in a fashion in which agrin would not bind α-DG with the same affinity as before.

### Agrin Exact Timing of Expression in the Human Heart

The regeneration potential of human cardiac muscular tissue is considered generally low, although a certain degree of cardiomyocyte renewal has been observed throughout life, with a peak reached at 10 years of age followed by a steady decrease throughout adulthood (Bergmann et al., 2015). The final number of CMs is reached already around 1 month after birth, but the exact timing (i.e., days or weeks) and molecular mechanisms by which the fully proliferative capacity of CMs would be substantially lost and replaced by a very low efficiency in cell division is unknown. No specific data have been collected so far on the transcription/expression levels of agrin or of other elements of the DGC within the first postnatal weeks, due to limitations imposed by obvious ethical concerns on the collection and use of human samples. Therefore, it is not known (i) how crucial is the role of agrin at different stages of human cardiac development and/or cardiomyocyte proliferation, (ii) whether exogenous agrin can exert on human hearts the same effect observed in mice. In this respect, some encouraging preliminary results have been obtained also in piglets, a model of MI more closely related to human cardiac physiology [see the preprint by Baehr et al. (2019)].

In order to fully grasp the possible therapeutic potential of agrin in heart regeneration, the levels of agrin in the human heart should be fully characterized as a function of time, from the embryonic to the childhood and through to the adult stage. This is undoubtedly a very challenging task, due to the limitations cited above that could, however, be at least partially overcome by analyzing (upon ethical approval and patient consent) peri-operative samples. Is agrin only expressed until early after birth, and then not expressed at all? In the light of what has been found in mice, it would be particularly important to collect data on the expression of agrin along all human developmental stages, which is a challenging task, mainly due to the intrinsic difficulties in collecting representative heart samples. Furthermore, is there a differential expression of agrin isoforms specific for each developmental stage? It seems likely that the A0B0 agrin isoform (the so-called muscle isoform, Gesemann et al., 1996) would be the most populated during development and neonatal growth, given that it has the largest affinity for α-DG, but this needs to be demonstrated yet. Ultimately, for a better therapeutic tailoring, it could be important to narrow down the expression profiles of other agrin splice isoforms in the human heart.

### “Switching on” an Agrin-Driven Specific Regenerative Program?

Agrin knockout in mice results in death at birth, but no gross histological abnormalities have been recorded in cardiac tissues (Gautam et al., 1996; Burgess et al., 2000). Similar findings have been described for the only human case reported of an agrin-null variant leading to perinatal death, (Geremek et al., 2020). Such evidence strongly indicates that agrin is not essential for CM proliferation during embryogenesis. This means that the DGC could well be involved in the sequestration of phosphorylated YAP at the cytosolic side of sarcolemma but, although agrin can induce DGC disassembly when administered to postnatal heart, factors other than agrin (or complementary to it) could induce the DGC disassembly believed to be responsible for YAP release during development. What is certain is that administration of exogenous agrin promotes the activation of this specific CMs proliferation program in mouse and possibly in larger mammals, as proposed by Baehr et al. in a very recent preprint manuscript (Baehr et al., 2019) reporting the regenerative effect of intracoronary injection of agrin into porcine models of myocardial infarction.

To the best of our knowledge, there is a lack of evidence to show that in larger mammals the regeneration mechanism induced by agrin is an established and conserved genetic program rather than a “*una tantum*” effect induced upon administration of exogenous protein. Again, a thorough investigation to prove whether a similar agrin-induced mechanism is in place also in human cardiac tissues, whether during development or/and exogenously induced, is granted.

### Translational Potential of Agrin for Heart Regeneration: Controlled Delivery of Agrin to the Damaged Tissues

From a translational point of view, a strategy based on an agrin-controlled proliferative program that can be triggered from outside CMs (i.e., within the ECM) should follow one of the basic principles of drug discovery and carefully consider the balance likely to exist between the beneficial and deleterious effects of an exogenous reactivation. In principle, any drug effective in raising the transcriptional levels of agrin could be appealing, but an estimate of the specific timing and dosage, as well of the most effective methods of administration, would be essential. If agrin is proven to be a competitor of laminin for binding to α-DG, important results could for example be obtained by reducing (temporarily) the expression of laminin. Another possibility would be to promote expression of a tailored agrin encompassing the α-DG binding site within the damaged tissues using retroviruses, that are commonly employed for delivering into cells nucleic acid sequences of interest to be integrated into the host cells genome. Indeed, a “mini-agrin” construct that binds to basement membranes as well as to α-DG has been already devised for use in skeletal muscle (Moll et al., 2001). Such construct is effective in attenuating the muscle pathology observed in a mouse model for congenital muscular dystrophy, through a mechanism that includes agrin-mediated stabilization of α-DG and the available laminin a5 chain. Retroviral strategies for a prolonged delivery of a series of therapeutic “mini-agrin” peptides (capable of binding both laminin and dystroglycan) have been recently proposed for the treatment of chronic pathologies such as congenital muscular dystrophies in available mouse model systems (Qiao et al., 2018; Yurchenco and McKee, 2019). In the case of agrin, “mini-agrin”, or truncated agrin delivery to diseased cardiac tissue, complementary strategies should be in place to make the re-activation highly controlled, as a prolonged delivery of agrin could induce undesired hyperproliferative effects.

Both in mouse (Bassat et al., 2017) and porcine (Baehr et al., 2019) models of myocardial infarction (MI), recombinant agrin injected locally in the damaged heart seems to have an overall cardioprotective effect, with anti-inflammatory and angiogenic components to add to CM protection and (mild) proliferation induction. As the authors suggest, agrin could then enhance heart healing *via* a series of pleiotropic effects, but the hypothesis that agrin may act through a promiscuous rather than a specific mechanism bears important implications/repercussions when trying to envisage regenerative strategies. As an example, administration of agrin has been shown to stimulate the production of blood vessels in both murine and porcine MI (myocardial infarction) models (Baehr et al., 2019), which draws an analogy with the recently discovered role of agrin in recruiting endothelial cells within tumors and its ability to overall promote tumor angiogenesis (Njah et al., 2019). Specifically, agrin has been found to act in endothelial cells (ECs) by an ECM stiffness based mechanism similar to that described in tumor cells, although the mechanotransduction axis would not involve the activation of YAP/TAZ but rather the stabilization of VEGFR2 (Vascular Endothelial Growth Factor Receptor 2), a master regulator of ECs migration, proliferation and angiogenesis. Whether agrin acts following a similar pathway also in cardiac angiogenesis is unknown, but since the agrin isoforms produced within growing tumors by cancer cells and ECs have a similar angiogenic effect (Njah et al., 2019), it could be speculated that this specific agrin axis could be the same acting in cardiac ECs. Paradoxically, even when administered locally, agrin could thus have beneficial effects on certain heart cells/tissues, but at the same time be dangerous for the surrounding ones not directly involved in the damage and healing process, where it could induce hyperproliferation. Considering the role of agrin in tumorogenesis and angiogenesis and the evidences collected on MI models, caution should be exercised when trying to envisage cardiac regenerative strategies involving this proteoglycan. In this respect, another crucial point to also consider in order to further assess the potential benefit of using therapeutic agrin would be to verify whether this protein is stimulating the hyperproliferation of human cardiac fibroblasts, or the activity of other factors involved in fibrotic tissue formation.

### Protein Therapy, Dosage, and Cost

The idea that an *in-situ* agrin-injection could potentially prevent or reduce the formation of scar tissue in the injured heart and stimulate the regrowth of healthy cardiac tissue is undoubtedly powerful. From an exquisitely biotechnological point of view, a full biochemical and structural characterization of the interaction of agrin with its partners primarily on CMs would be fundamental for the optimization of a series of parameters typically considered in drug discovery, such as (i) the solubility and stability of the employed recombinant protein, (ii) its capacity to trigger the regenerative program with high specificity, and thus (iii) the most effective dosage (with the least adverse effects), (iv) the final cost of treatments involving a recombinant system for the production of full-length agrin, or of the agrin C-terminal peptides that harbor the α-DG binding site. To note, as an alternative to local injection more physiological methods of administration could be employed. A promising example is offered by cardiac patches made of natural materials with specific biodegradable and biocompatible properties emulating closely the myocardial microenvironment (Albertario et al., 2019; McMahan et al., 2020), to be implanted upon corrective cardiac surgery. Such patches could be used as a matrix for storage and controlled release of agrin with different modalities. As an example, collagen scaffolds have been used to deliver vascular endothelial growth factor (VEGF) either by modifying the protein to include a collagen-binding domain or by seeding the scaffold with cells that have been genetically engineered to express VEGF, thereby extending the delivery time and promoting vascular growth in infarcted myocardial tissue [reviewed by Ye et al. (2013)]. The last frontier would be that of employing the very latest technologies that created an acellular cardiac patch composed of synthetic microparticles containing secreted cardiac regenerative factors embedded in a scaffold formed by decellularized myocardial ECM (Huang et al., 2020), to allow release of agrin in the absence of the complications associated with cellular components. However, it remains to be determined whether any of the above patches would allow for the necessary time-restricted controlled delivery of recombinant agrin to the damaged heart, and local injection might still be the preferential and more efficient mean of administration, as recently shown in pig models of MI (Baehr et al., 2019).

## Conclusions

Agrin might represent a new player in the race to find innovative treatments for the cure of damaged hearts. Although the preliminary results obtained show great promise, it is still too early to foresee whether exogenous agrin-induced heart regeneration could become a realistic therapeutic avenue for injured human cardiac muscle. A thorough investigation at the molecular level into the proposed mechanism and a characterization of the role played by all factors involved will be pivotal to fully uncover the translational potential of this regenerative pathway.

## Author Contributions

MB and AB conceived the idea and wrote the first draft of the manuscript. KS and FJ prepared the illustration and box. KS, FJ, and MC reviewed and contributed to the various draft versions of the manuscript. All authors read and approved the final manuscript.

## Conflict of Interest

The authors declare that the research was conducted in the absence of any commercial or financial relationships that could be construed as a potential conflict of interest.
