# Corrigendum: Agrin-Mediated Cardiac Regeneration: Some Open Questions

**DOI:** 10.3389/fbioe.2020.00935

**Published:** 2020-08-21

**Authors:** Maria Giulia Bigotti, Katie L. Skeffington, Ffion P. Jones, Massimo Caputo, Andrea Brancaccio

**Affiliations:** ^1^Bristol Heart Institute, Research Floor Level 7, Bristol Royal Infirmary, Bristol, United Kingdom; ^2^School of Biochemistry, University of Bristol, Bristol, United Kingdom; ^3^Institute of Chemical Sciences and Technologies “Giulio Natta” (SCITEC)—CNR, Rome, Italy

**Keywords:** heart regeneration, agrin, dystrophin-glycoprotein complex, dystroglycan, laminin, YAP, Hippo pathway, cardiomyocyte proliferation

There is an error in the Funding statement. The correct number for the British Heart Foundation funding is grant no. CH/1/32804.

The authors apologize for this error and state that this does not change the scientific conclusions of the article in any way. The original article has been updated.

